# Performance Analysis of Three Side Roughened Solar Air Heater: A Preliminary Investigation

**DOI:** 10.3390/ma15072541

**Published:** 2022-03-30

**Authors:** Aruna Kumar Behura, Chinmaya Prasad Mohanty, Manas Ranjan Singh, Ashwini Kumar, Emanoil Linul, Dipen Kumar Rajak

**Affiliations:** 1 School of Mechanical Engineering, Vellore Institute of Technology, Vellore 632014, TN, India; akbehura.nit@gmail.com (A.K.B.); chinmaymohantymech@gmail.com (C.P.M.); 2 Department of Mechanical Engineering, Silicon Institute of Technology, Bhubaneswar 751024, OD, India; manasranjan.singh@gmail.com; 3 Department of Mechanical Engineering, Shree Guru Gobind Singh Tricentenary University, Gurugram 122505, HR, India; aknitjsr08@gmail.com; 4 Department of Mechanics and Strength of Materials, Politehnica University Timisoara, 300222 Timisoara, Romania; 5 Department of Mechanical Engineering, G. H. Raisoni Institute of Business Management, Jalgaon 425002, MH, India

**Keywords:** collector efficiency factor, collector heat removal factor, Reynolds number, pitch of relative roughness, height of relative roughness, mass flow rate

## Abstract

In recent years, sunlight has been used in several fields such as photovoltaic cells, flat plate collectors, solar cookers, green buildings, and agricultural applications. Improved thermal performance has been seen which comes of three sides absorber plate with glass cover compared to the traditional one. This paper presents the Nusselt (Nu) number, collector efficiency factor (CEF), and collector heat removal factor (CHRF) for the optimal solution of three sides artificially roughened solar air heater. Five input variables such as Reynolds (Re) number, relative roughness pitch, relative roughness height, mass flow rate, and air temperature of the duct are taken into account for improved efficiency optimization of collector, collector heat removal factor, and Nu number. Technique for order of preference by similarity to ideal solution (TOPSIS) technique is used to identify the best alternative amongst a number of performance measures by converting them into an equivalent single variable. Moreover, the results revealed the high accuracy of the CEF, CHRF, and Nu number of 75–80%, 74–78%, and 63–71%, respectively. Meanwhile, it has been also observed that roughness Re number varies between 12,500 and 13,500, and height of relative roughness is 0.0245, including pitch of relative roughness 10 along with the rate of mass flow is 0.041 kg/s.

## 1. Introduction

Sunlight is the major source of renewable energy which provides heat energy without any pollution. Solar Air Heaters (SAHs) play a vital role in the conversion of sunlight into heat energy or thermal energy, which can be utilized for many thermal applications in day-to-day life. Utilization of additional roughness in the collector plate of a solar collector provides better efficiency as compared to the smooth one reported by many researchers. Collector heat removal factor is defined as the quantity that relates the actual useful heat gain of a collector to the useful heat gain, if the whole collector surface were experienced to air inlet temperature, while, Collector Efficiency Factor is the ratio of actual useful energy gain to the useful energy gain of the absorber plate [1]. The addition of roughness in the collector of a solar channel provides increment in collector heat removal factor (CHRF) and Collector Efficiency Factor (CEF), which plays a vital role in enhancing the thermal efficiency of a SAH. It has been reported that better thermal efficiency can be achieved using unevenness in the collector of a single side SAH [2]. The utilization of optimized artificial roughness in SAH to improve thermo-hydraulic performance (THP) has been illustrated by Prasad and Saini [3]. A unique design for counter flow curved double pass solar collector has been proposed by Kumar et al. [4] to show its performance characteristics and to be compared with various parallel designs under different flow and geometric conditions. The impact of artificial roughness on the performance of SAHs has been studied in [5] to provide comprehensive information about the different cross-sectional roughness elements, and the effect of these roughness elements on the performance of SAHs has been discussed. The different directions and arrangements have been studied by Saurav and Sahu [6] for artificial roughness in SAHs. Furthermore, the utilization of various artificial roughness geometries has been reported in SAHs to enhance the heat transfer rate and thermal effectiveness [7]. A study of Computational Fluid Dynamics (CFD) for the Y-shaped wire utilized as an unnatural roughness in SAHs for enhancing the heat transfer rate was conducted by Singh et al. [8]. In addition, the CFD code and interrelation for rib unevenness of equilateral triangular and square-sectioned transverse utilized on the collector of solar air collectors have been reported by Yadav and Bhagoria [9,10]. Moreover, Gawande et al. [11] analyzed the effect of various roughness geometries on heat transfer enhancement in different solar thermal systems. Experimental studies of heat transfer and thermal performance with longitudinal fins of SAH have also been reported [12].

The literature studied above mentioned only one side artificial roughness SAHs. A SAH duct with three artificially roughened sides (the two side walls and the top side) has been analyzed, investigated, and optimized by Prasad et al. [13,14], for more increase in THP than in one side only. It has been reported that the use of the inside number of fin array in SAH enhances the performance variables [15,16]. Using numerical analysis, Singh [17] evaluated the behavior of a SAH with an arched absorber plate. The author observed a significant improvement in the THP of the proposed system. The value of transfer of heat is reported in [18,19,20] for one side and three sides artificial roughness SAH using the Artificial Neural Network technique. Abuska [21] performed an analysis on a new SAH with the conical surface and compared the results with a flat absorber plate. He noted that the conical elements ensure a smooth flow of air above the absorbent plate, an increased surface area, relatively less shading, and a decrease in the dead surface of the duct. As a result, there is a prominent increase in thermal efficiency for the conical type compared to a flat one. The thermal performance of a double pass SAH with and without turbulators has been studied experimentally by Abdullah et al. [22]. The authors obtained that the double pass SAH shows improvements in the daily efficiency of up to 30% compared to a single pass SAH. Investigating the performance of different SAHs with jet impingement [23], natural and forced convection modes [24] and landscape fabric [25] has gained major interest in recent years. Furthermore, the effects of wavelength and amplitude [26], and the different fins shape [27] on the performance of SAHs were of interest to researchers. The thermal performance enhancement of SAH with a rugged plane collector was investigated by Ansari and Bazargan [28]. The authors conducted an optimization study both to obtain higher efficiency and to guarantee an appropriate temperature difference between inlet-outlet airflow. Singh and Singh [29,30] studied the effect of various relative height and pitch ratios of V-groove corrugation and semicircular groove on the dynamic THP of curved SAHs. Jia et al. [31] investigated the performance (including temperature differences between inlet and outlet, heat collection efficiency, air volume, and irradiance) of the spiral SAH for different weather conditions. The authors found that, compared to conventional and serpentine SAHs, this type of SAH has a higher heat collection efficiency. A v-groove and flat plate were developed in [32] to be used in SAHs. The authors noted that the heat transfer coefficient and instantaneous energy efficiency of the SAH were higher in the case of the v-shaped groove air heater. The THP characteristics of a SAH roughened by multiple v-shaped ribs were also investigated in detail [33,34]. It was reported that the main geometrical parameters of the channel and rib affect the optimal span-wise rib number. A mathematical model established [35] for the coefficient of transfer of heat and friction factor of collector having twisted roughened SAH to improve the restriction of performance. A v-shape configuration investigated [36] SAH and also developed the mathematical model for friction factor and Nusselt (Nu) number. Saravanakumar et al. [37] observed that the use of an arc-shaped rib roughened SAH integrated with fins and baffles improves the efficiency and effectiveness. The utilization of multi V-rib and prominent component reported [38] on the solar air collector for the increment of THP and Nu number. The utilization of the number of gaps analyzed in arc type roughened SAH for enhancement of heat transfer coefficient [39]. The utilization of w-type roughened SAH developed [40,41] accompanied by similar distance on the channels for improvement of Nu number with an additional effect of booster mirrors. Similarly, a number of works have been reported in the most recent years to show the extensive improvements in exergy, CFD, and THP of solar air collectors by using different methodologies [42,43,44,45,46,47,48].

An extensive study of past literature directs that a good amount of work has been reported to improve the performance of solar air heaters such as one side artificial roughened solar air heater with various geometries of roughness, three sides artificial roughened solar air heater with transverse, w-shaped, v-shaped roughened absorber plate, etc. However, process optimizations of the solar air heaters are not given adequate importance in the area of solar energy. Selection of optimum process condition is reported [49] to improve the performance of solar air heaters, the optimization of performance parameters of three side’s rugged solar air heater. Mostly, researchers have optimized and analyzed the performance measures such as optimization of process variables such as Nu number, friction factor, roughened Reynolds (Re) number, and thermal efficiency. Therefore, there exists to propose an efficient optimization framework for the optimization performance measures such as collector heat removal factor, collector efficiency factor, Nu number to improve the performance of a solar air heater. Therefore, to address the above-discussed literature gap, the authors have attempted to optimize the process parameters of solar air heater viz. ambient temperature, Re number, mass flow rate, relative roughness height, relative roughness pitch for the best performance of three important performance measures such as collector heat removal factor, collector efficiency factor, and Nu number, while keeping friction factor minimum with less pumping power. The multiple responses are transformed into an identical sole performance measure closeness factor identifying the best-ranked solution from the experiments. Finally, the best-ranked solution is further improved by a hybrid optimization technique TOPSIS (a technique for order of preference by similarity to ideal solution) based Cuckoo Search Optimization (CSO) technique. The technique is easy in concept, easy for execution, and converges rapidly to achieve the optimum solution, which sets the approach apart from other techniques [50,51,52]. The experimental investigation of bioconvection Casson nanofluid flow using the Darcy–Forchheimer 3D technique on a whirling disc is reported [53]. Recently, researchers have used numerous Multi-Criteria Decision-Making (MCDM) tools for selecting the best alternative among the available performance measures by converting them into a corresponding single performance measure. TOPSIS is one such technique that uses the principle that the selected alternative certainly be nearest to the positive best solution and must be farthermost from the negative best solution [54,55]. In comparison to other approaches, the method is easy and requires fewer statistical calculations. The optimum process condition acquired utilizing this mixed technique is certified by conducting a supporting trial. This system provides inspiration to researchers and industrialists to use customized systems for their applications such as crop drying, fish drying, storage of heat energy, etc.

## 2. MCDM-TOPSIS Approach

The TOPSIS method is presented stepwise underneath.

▪ ***First step*:** In this study, the responses such as Nusselt number (Nu), collector heat removal factor, and collector efficiency factor are taken as favorable attributes. So, attempts are made to maximize these parameters. Based on the equation provided below the response matrix is standardized.
  (1) $$Q_{\mathrm{ij}}=\frac{b_{\mathrm{ij}}}{\sqrt{\sum_{i=1}^{m}b_{\mathrm{ij}}^{2}}}$$
  where i = 1…. m and j = 1…. n., b_ij_ represents the actual ith value of jth experiment and Q_ij_ represents the correlated standardized value.
▪ ***Second step*:** Equal and uniform weight is assigned to all three objectives, hence the weighted normalized matrix, is assigned at 0.33. This weight is multiplied with the normalized matrix obtained through Equation (1); therefore the relation is given by Equation (2).
  U_ij_ = W_i_ × Q_ij_ (2)
  where U_ij_ is the weighted normalized matrix and W_i_ marks the weight of the jth attribute.
▪ ***Third step:*** The positive best result (PBR) and negative best result (NBR) are estimated using Equations (3) and (4).
  X^+^ = (X_1_^+^, X_2_^+^, X_𝑛_^+^) for all the upper values (3)
  X^−^ = (X_1_^−^, X_2_^−^, X_𝑛_^−^) for all the lower values (4)
▪ ***Fourth step:*** The positive best result (PBR) and negative ideal solution (NIS) are estimated by utilizing the following equations.
  (5) $${\;T}_{i}^{+}=\sqrt{\overset{m}{\underset{j=1}{\sum }}{\left(x_{\mathrm{ij}}-x_{j}^{+}\right)}^{2}}$$
  (6) $${\;T}_{i}^{-}=\sqrt{\overset{m}{\underset{j=1}{\sum }}{\left(x_{\mathrm{ij}}-x_{j}^{-}\right)}^{2}}$$
  where i = 1, 2 … n.
▪ ***Fifth step:*** The following relation is used to estimate the closeness factor (CF)
  (7) $$\mathrm{CF}=\frac{T_{i}^{-}}{T_{i}^{+}+T_{i}^{-}}$$

The closeness factor CF ranks the alternatives from ideal to worst. The alternative having the highest order of closeness factor is identified as the best alternative among the alternatives.

## 3. Experimental Strategy

As per the literature survey, mass flow rate, height of relative roughness, pitch of relative roughness, Re number, and atmospheric temperature are picked as the major process variables [56]. The collector efficiency factor, collector heat removal factor, and Nusselt number are scrutinized as principal performance quantities in the current research. The process parameters and their codes are provided in Table 1.

In this work, experiments are planned as per Box–Behnken design of response surface methodology (RSM) as the process executes non-sequential experiments having less number of design points. It works in a safe operating zone as it does not have any axial point. However, central composite designs possess axial points outside the cube which does not operate properly and falls beyond the safe operating area. [57,58]. Table 2 represents the Box–Behnken design along with obtained responses. The parameters are identified based upon a literature survey and hence decided to study their outcomes on process measures viz. collector efficiency factor, collector heat removal factor, and Nusselt number by using response surface methodology (RSM) Box–Behnken design.

Table 2 shows the RSM Box–Behnken design of forty-six trial runs accompanied by the performance estimations viz; CHRF, CEF, and Nu number. The multiple responses (CHRF, CEF, and Nu number) are transferred to its identical response and factor of closeness utilizing the TOPSIS technique. Table 2 also represents the finest ranked experimental outcomes out of the 46 trial runs secured after the application of the TOPSIS technique. The investigational trial (3 times) executes the elevated order of 0.919987 out of all the execution quantities.

## 4. Results and Discussions

In this work, the Box–Behnken design of response surface methodology (RSM) is adopted to plan the experiments as it conducts non-sequential trials and possesses lesser design points. The technique is proficient enough to draw out maximum information from the study by operating in the safe zones with no axial points [57,58]. Forty-six trial runs were performed based on Box–Behnken design to evaluate the effect of mass flow rate, relative roughness pitch, ambient temperature, Re number, and relative roughness height on responses. ANOVA stands for Analysis of Variance, the technique is proficient enough to identify and quantify the most influential parameter amongst number of variables. [57,58]. Table 3 shows the ANOVA table for collector efficiency factor, it can be seen that at the significance level of 0.5, all the parameters are having a significant effect on the collector efficiency factor. Similarly, from Table 4 it can be seen that mass flow rate, relative roughness pitch, Re number, and ambient temperature are the important process parameters for the collector efficiency factor. Again, from Table 5 it can be seen that all the process parameters have a significant effect on the Nu number. Table 6 shows the ANOVA for the closeness factor. From the table, it can be seen that mass flow rate, relative roughness height, relative roughness pitch, ambient temperature, Re number are having significant effects on the variation of closeness factor. Apart from this interaction of mass flow rate × Re number, mass flow rate × Ambient temperature, relative roughness pitch × Re number, and square terms of mass flow rate, relative roughness pitch are found to be the important process parameters for closeness factor.

**Figure 1:**
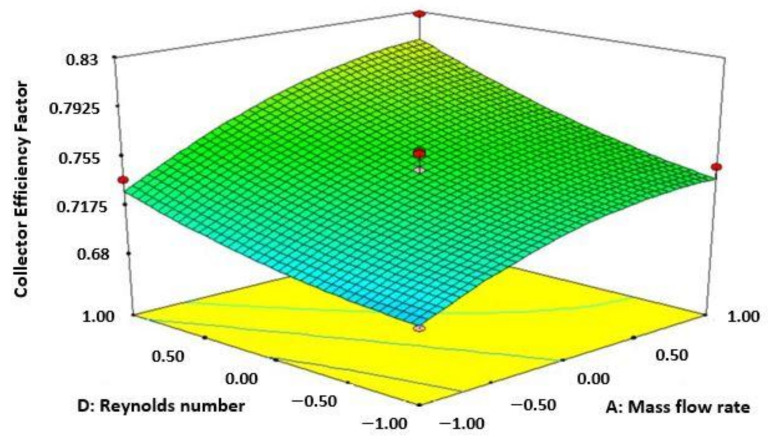
Variation of collector efficiency factor (Re number and mass flow rate).

Figure 1 shows the increase of collector efficiency factor (CEF) accompanied by Re number and rate of mass flow. This can be seen from the diagram that enhancement of CEF with increasing values of both Re number and mass flow rate for solar collector of 3 sides absorbing capacity. The diagram also shows that the highest value of CEF is attained at the mass flow rate of 0.04 m/s and Re number of 13,000. The purpose of attributes used in this study creating a fully developed turbulent flow inner part of the absorber plate or inside the duct which results in an increase in the CEF in terms of rate of heat transfer and also collector leading to enhancement in Nu number [10].

**Figure 2:**
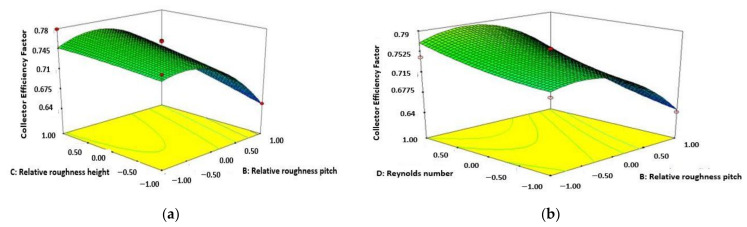
Variation of collector efficiency factor (Relative roughness pitch and relative roughness height) (**a**); variation of collector efficiency factor (Re number and relative roughness pitch) (**b**).

Figure 2a represents the enhancement of CEF with respect to the increasing value of height of relative roughness and decreasing amount of pitch of relative roughness. The diagram shows that the collector effectiveness factor enhances accompanied by the increasing amount of relative roughness height and decreasing value of relative roughness pitch. This can be noticed that the maximum functionality of CEF is attained accompanied by the maximum value of relative roughness height as 0.0245 and the least functionality of relative roughness pitch as 10. With the increasing value of relative roughness pitch from 10 to 20, the turbulence at the inner part of the collector reduces which results in a decrease in temperature at the exit. As a result, the CEF reduces with enhancement in relative roughness pitch. The value of CEF enhances accompanied by improvement of Re number and decreasing amount of pitch of relative roughness shown in Figure 2b. The highest amount of CEF attained from the diagram at the value of Re number as 13,000 and relative roughness pitch as 10.

**Figure 3:**
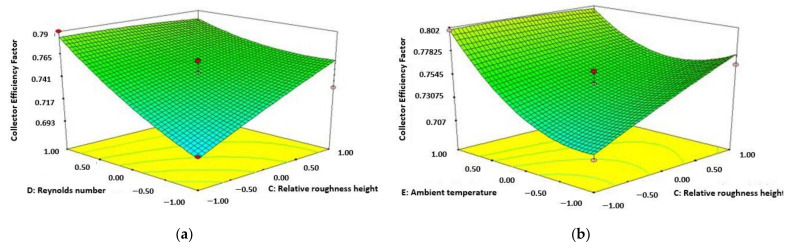
Variation of collector efficiency factor (Re number and relative roughness height) (**a**); variation of collector efficiency factor (ambient temperature and relative roughness height) (**b**).

Figure 3a shows the amount of CEF increases with the enhancing amount of both height of relative roughness and Re number. The maximum amount of CEF achieved from the diagram at the amount 13,000 for Re number and 0.0245 for height of relative roughness. Figure 3b represents the value of CEF enhancement accompanied by the improving amount of height of relative roughness and ambient temperature. The parameters are coded in 3 levels such as −1 is the lower level, +1 is the highest level and 0 is the medium level.

**Figure 4:**
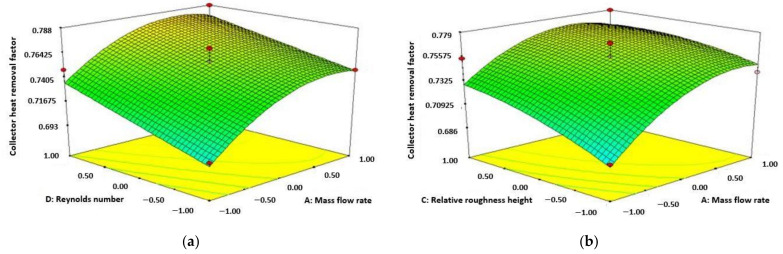
Variation of collector heat removal factor (Re number and mass flow rate) (**a**); variation of collector heat removal factor (relative roughness height and mass flow rate) (**b**).

Figure 4a shows the increase of collector heat removal factor (CHRF) with Re number and mass flow rate. This can be noticed from the diagram that increment of CHRF accompanied by increasing values of both Re number and mass flow rate for solar collector of 3 sides absorbing capacity. The diagram also shows that highest value of CHRF is achieved at the mass flow rate of 0.041 m/s and Re number of 13,000. The purpose of attributes used in this study creating a fully developed turbulent flow inner part of the absorber plate or inside the duct which results in increases in the CHRF in terms of rate of heat transfer and also collector directing to increase in Nu number. Figure 4b represents the enhancement of CHRF with respect to the increasing value of both relative roughness height and rate of mass flow. The figure represents that CHRF enhances accompanied by improving the amount of both mass flow rate and height of relative roughness. It can be observed that the maximum functionality of CHRF is attained with the highest amount of relative roughness height as 0.0245 and mass flow rate as 0.04 m/s. With the increasing value of relative roughness height from 0.0135 to 0.0245, the turbulence at the inner part of the collector reduces which results in the decrease of temperature at the exit. As a result, CHRF reduces accompanied by enhancing the pitch of relative roughness.

**Figure 5:**
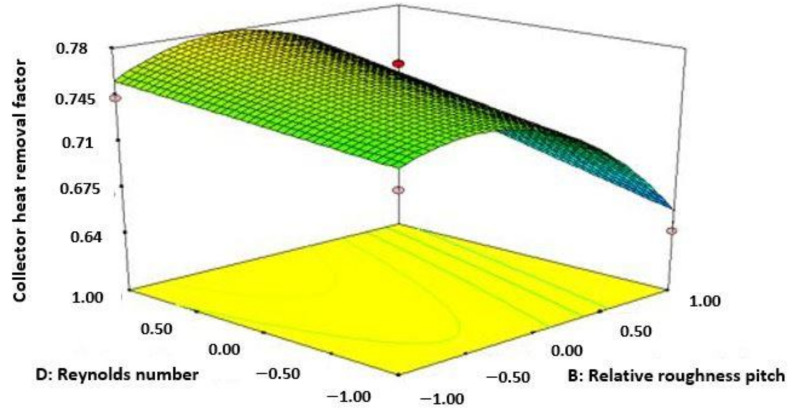
Variation of collector heat removal factor (Re number and relative roughness pitch).

Figure 5 represents the amount of CHRF increases accompanied by the increase of Re number and reducing the value of relative roughness pitch. The highest amount of CHRF was attained from the diagram at the amount of 13,000 for Re number and 10 for pitch of relative roughness.

**Figure 6:**
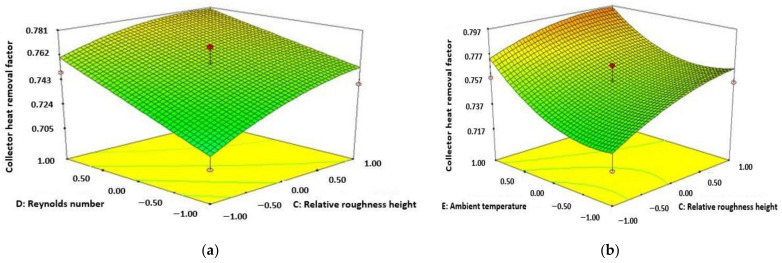
Variation of collector heat removal factor (Re number and relative roughness height) (**a**); variation of collector heat removal factor (ambient temperature and relative roughness height) (**b**).

Figure 6a shows that the value of CHRF improving accompanied by enhancing the amount of both height of relative roughness and Re number. The maximum amount of CHRF achieved from the diagram at the amount of 13,000 for Re number and 0.0245 for the height of relative roughness. Figure 6b represents the value of CHRF enhancement accompanied by improving the amount of height of relative roughness and ambient temperature.

**Figure 7:**
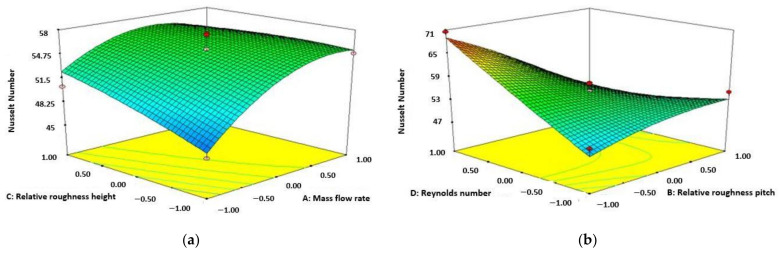

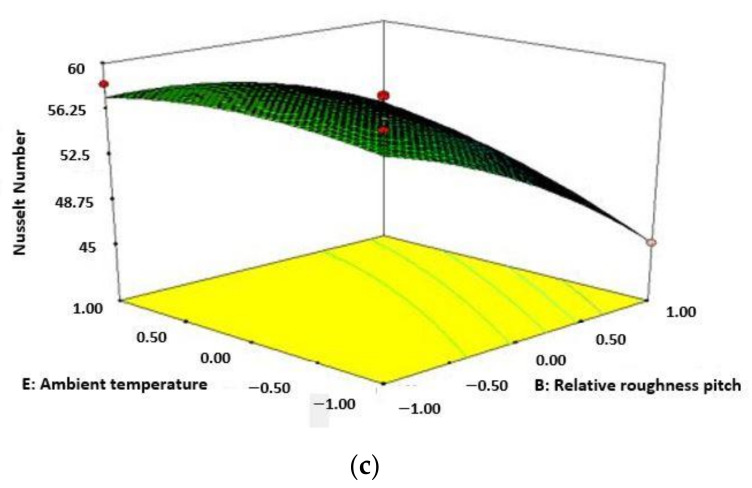
Variation of Nu number (relative roughness height and mass flow rate) (**a**); variation of Nu number (Re number and relative roughness pitch (**b**); variation of Nu number (ambient temperature and relative roughness pitch) (**c**).

Figure 7a shows the increase of Nu number with relative roughness height and mass flow rate. It can be seen from the figure that enhancement of Nu number with an increasing amount of height of relative roughness and mass flow rate for the solar collector of 3 sides absorbing capacity. The diagram also represents the maximum amount of Nu number is achieved at the mass flow rate of 0.04 m/s and height of relative roughness of 0.0245. The purpose of attributes used in this study creating a fully developed turbulent flow inner part of the absorber plate or inside the duct which results increase the Nu number in terms of rate of heat transfer and also the collector leading to an increase in CHRF.

Figure 7b represents the enhancement of Nu number with respect to the increasing value of Re number and decreasing value of relative roughness pitch. The diagram shows the Nu number enhances with improving value of Re number and reducing the amount of pitch of relative roughness. This can be observed that the maximum functionality of Nu number is attained with the maximum amount of Re number as 13,000 and the least functionality of pitch of relative roughness as 10. With the increasing value of pitch of relative roughness from 10 to 20, the turbulence at the inner part of the collector reduces which results in a decrease of temperature at the exit. Figure 7c represents the value of Nu number improves accompanied by enhancement of ambient temperature and reducing the value of relative roughness pitch.

In the current work, five process parameters are considered for the study, i.e., mass flow rate, height of relative roughness, Re number, ambient temperature, and pitch of relative roughness. Shortly, after experimentation the three performance measures considered in the study such as factor of collector efficiency, factor of collector heat removal and Nu number are transformed into a similar closeness factor (CF) by means of the TOPSIS approach. Further, the five process parameters as Re number, relative roughness pitch, relative roughness height, mass flow rate, and air temperature are related to the closeness factor by means of non-linear regression analysis. Furthermore effort is made to maximize the value of the closeness factor by using the Cuckoo Search (CS) algorithm. Hence, the target purpose of CF is composed as given by Equation (8).

Closeness factor = +0.41 − 0.11 × A + 0.15 × B − 0.057 × C − 0.11 × D − 0.080 × E − 1.740 × 10^−3^ × A × B + 0.036 × A × C − 0.10 × A × D + 0.090 × A × E − 0.049 × B × C+ 0.095 × B × D − 0.046 × B × E + 0.034 × C × D−0.013 × C × E − 0.021 × D × E + 0.12 × A^2^ + 0.19 × B^2^ + 0.038 × C^2^ + 4.156 × 10^−3^ × D^2^ + 8.388 ×10^−3^ × E^2^ (8)

### 4.1. Proposed Cuckoo Search

A hybrid optimization model has been proposed to enhance the performance by using the TOPSIS multi-criteria algorithm and Cuckoo Search Optimization (CSO) Algorithm. CSO algorithm is widely used in various fields of engineering problems [50,51,57] due to its exploration and exploitation ability. In this work, TOPSIS-CSO is used to optimize the critical parameters to test the effectiveness of three side roughened solar air heater performance.

Yang and Deb in 2009 [50] developed a meta-heuristic algorithm based on the natural behaviors of the cuckoo bird on the concept of laying their eggs in a communal bird’s nest. Cuckoos generally lay their fertilized eggs in other cuckoos’ nests with the hope of their off-springs being raised by proxy parents. The cuckoos have to take a decision to throw the foreign eggs out of the nests or the whole nests to abandon and build a new nest elsewhere when they discover that the eggs in their nests do not belong to them. The possible solution to the problem is the bird nest having each egg suggestions and a new better solution is indicated by the cuckoo’s egg.

▪ The cuckoo search optimization algorithm depends on three conditions.
▪ Every cuckoo lays a single egg at a time and throws its egg in a random nest.
▪ The high quality of eggs which is considered as the best solution in the best nest will carry forward to the next generation.
▪ The host bird is exposed to the alien egg with a probability (Pa ∈ (0, 1)).

A new random walk search technique was substituted by Levy flight to obtain a better solution [48].

The new solution $\left(X_{t+1}\right)$ is obtained by the use of Levy flight for the cuckoo bird by Equation (9):

(9) $$X_{\left(t+1\right)}^{i}=X_{\left(t\right)\;}^{i}+\propto \;⊕\lambda$$

where t signifies the happening iteration, ∝ is the fixed step size as per the problem dimension (∝ > 0), ⊕ denotes the entry-wise operation of multiplication, and the Levy fight parameter is denoted as λ.

Levy fight is based on levy distribution for the step length of a random walk. In an uncertain environment, Levy fights are capable to achieve maximum efficiency [49]. The Levy distribution is expressed as

(10) L s,ϑ,γ=ϑ2πexp−ϑ2s−γ1s−γ32  0<γ<s<0              0                                                           Otherwise       

where γ > 0 a minimum is step and mostly it is considered as 1 and ϑ is a scale parameter.

The step length s can be calculated with Equation (11):

(11) s=uv1∅

here ∅ is an index, ranging between 1 ≤ ∅ ≤ 2.

From normal distribution u and v are obtained as follows:

(12) $$u\sim \;N\;\left(0,\sigma_{u}^{2}\;\right)$$

(13) $$v\sim \;N\;\left(0,\sigma_{v}^{2}\;\right)$$

Normal distribution (N), $\sigma_{u}$ and $\sigma_{v}$ are as follows:

(14) σu=Γ1+∅sin(π∅/2)Γ1+∅2∅ 21+∅21∅, σv=1

Here the gamma function (Γ) is:

(15) $$\Gamma \left(z\right)=\int_{0}^{\infty }t^{z-1}e^{-t}\;\mathrm{dt}$$

### 4.2. Proposed Cuckoo Search with Levy Flights Approach

**Figure 8:**
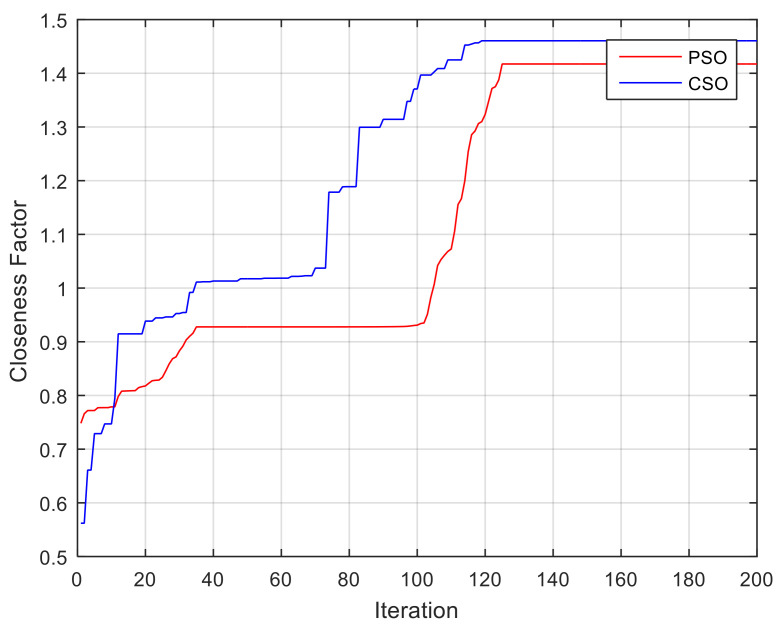
Convergence curve for CSO and PSO algorithms. CSO = 1.4605 (−1, 1, −1, 1, −1)

The algorithm is coded in Matlab in core i7 processor to evaluate the potential of the algorithm. This resulted in the convergence of the optimum solution. To validate the proposed CSO algorithm we have compared the obtained results with another popular algorithm such as PSO. Figure 8 is distinguished that the CSO algorithm results are better than PSO as the best solution is converged rapidly. After 120 iterations, it can be observed that the value of the closeness factor is obtained by CSO and PSO is 1.46 and 1.41, respectively.

To check the predicted results of the solutions acquired through the hybrid optimization (TOPSIS based CSO) approach, yet again a test is conducted on the optimum results. Immediately after the test the performance measures viz. heat removal factor, collector efficiency factor, and Nu number are measured. Then, the closeness factor (CF) is estimated and it is perceived that the value of CF is improved by 8.58% when compared with the previous best obtained from the TOPSIS approach (Table 2). The confirmative test outcomes of CF along with the optimum process parameters and the improved value of the CF are given in Table 7. The Algorithm 1 shows the CSO algorithm.
**Algorithm 1:** Cuckoo Search Optimization (CSO).***Start*** Objective function f(x) Random generation of initial population of n host nests xi (i = 1, 2, …, n) ***While*** (t < Max Generation);   Get a cuckoo randomly by Levy flights;   Evaluate its fitness F(i);   Choose a nest among n (Let the nest, j) randomly; $\mathrm{if}\;(\;F^{i}>F^{j}$ The new solution is to replace j; ***end*** A probability (pa) of worse nests are unrestrained and a new nest is constructed; The best solutions are stored, i.e., the nests having the quality solution Solutions to be ranked and find the current best; ***end while*** ***end*** ***Result***

## 5. Conclusions

A considerable investigation and multi-criteria growth of operational variables of three side rugged solar air heater (SAH) utilizing a novel multi-criteria decision-making methods (MCDM) algorithm have been represented in this research. TOPSIS (technique for order of preference by similarity to ideal solution) technique is used in this work that provides accurate values, which help solar engineers for designing the better collector without simulation. Additionally, the better result is recognized through the MCDM approach is certified through regulating an approving examination forecasting outcome of approximately 8.58% of improvement as compared to the existing one. The conclusions that can be drawn from the investigational analysis are as follows:

Pitch of relative roughness and Reynolds (Re) number have played a vital role for all the output parameters, while, collector efficiency factor (CEF), collector heat removal factor (CHRF), and Nusselt number, relative roughness height has a minimum role for the variation of results. The maximum value of collector efficiency factor (CEF), is found to be 75–81 when the value of relative roughness height is 0.0245, relative roughness pitch is 10, Reynolds number is about 12,500–13,000, and the mass flow rate is 0.041 kg/s. When the value of the outlet temperature, the Reynolds number, and the ambient air temperature are increased and the relative roughness pitch decreases, the value of thermal efficiency improves. The maximum range of collector heat removal factor is noted to be 74–78%, while the scale of Re number is approximately 12,500–13,000, the amount of pitch of relative roughness is 10, range of height of relative roughness is approximately 0.0245 and the value of mass flow rate is 0.041 kg/s. The maximum range of Nu number are noted to be 63–71%, while the value of Re number is about 12,500–13,000, the value of relative roughness height approximately 0.0245, relative roughness pitch of 10, and the mass flow rate is 0.041 kg/s. This system inspires the use of custom systems for applications such as heat storage, crop drying, fish drying, etc.

## Figures and Tables

**Table 1:** Process parameters and their levels.

Process Parameters	Unit	Symbols	−1	Levels	1
Mass flow rate	Kg/s	A	0.011	0.0175	0.0248
Pitch of relative roughness	mm	B	10	15	20
Height of relative roughness	mm	C	0.0135	0.0225	0.0247
Re number		D	5000	10,000	13,000
Atmospheric temperature	°C	E	27	30	32

**Table 2:** RSM Box–Behnken experimental design along with obtained responses.

Sl. No	A	B	C	D	E	Collector Heat Removal Factor	Collector EfficiencyFactor	Nusselt Number	Closeness Factor (CF)
1	−1	−1	0	0	0	0.698	0.692	50.9	0.730813
2	1	−1	0	0	0	0.745	0.755	55.8	0.514159
3	−1	1	0	0	0	0.631	0.639	43.7	0.919987
4	1	1	0	0	0	0.685	0.699	48.8	0.776373
5	0	0	−1	−1	0	0.705	0.695	50.2	0.669547
6	0	0	1	−1	0	0.74	0.73	54.9	0.498939
7	0	0	−1	1	0	0.749	0.789	57.4	0.371548
8	0	0	1	1	0	0.805	0.755	60.2	0.338904
9	0	−1	0	0	−1	0.749	0.744	59.1	0.454069
10	0	1	0	0	−1	0.669	0.665	45.1	0.911269
11	0	−1	0	0	1	0.791	0.799	58.3	0.37341
12	0	1	0	0	1	0.729	0.725	51.9	0.648318
13	−1	0	−1	0	0	0.689	0.679	45.9	0.796169
14	1	0	−1	0	0	0.748	0.741	54.9	0.486216
15	−1	0	1	0	0	0.754	0.714	50.4	0.600135
16	1	0	1	0	0	0.779	0.799	53.2	0.433297
17	0	0	0	−1	−1	0.77	0.76	46.8	0.604707
18	0	0	0	1	−1	0.795	0.785	54.1	0.416158
19	0	0	0	−1	1	0.791	0.781	47.9	0.546663
20	0	0	0	1	1	0.819	0.869	57.1	0.274772
21	0	−1	−1	0	0	0.741	0.747	56.5	0.50565
22	0	1	−1	0	0	0.699	0.649	44.1	0.875914
23	0	−1	1	0	0	0.759	0.779	57.1	0.448278
24	0	1	1	0	0	0.702	0.722	54.1	0.624424
25	−1	0	0	−1	0	0.696	0.686	52.1	0.641054
26	1	0	0	−1	0	0.741	0.748	49.1	0.601537
27	−1	0	0	1	0	0.748	0.738	50.9	0.570786
28	1	0	0	1	0	0.788	0.828	66.3	0.114451
29	0	0	−1	0	−1	0.717	0.707	53.9	0.563956
30	0	0	1	0	−1	0.755	0.765	52.1	0.510247
31	0	0	−1	0	1	0.759	0.799	57.2	0.380239
32	0	0	1	0	1	0.783	0.793	60.3	0.275463
33	−1	0	0	0	−1	0.709	0.699	43.1	0.783184
34	1	0	0	0	−1	0.741	0.745	58.5	0.404511
35	−1	0	0	0	1	0.719	0.749	57.2	0.446054
36	1	0	0	0	1	0.791	0.799	53.1	0.427346
37	0	−1	0	−1	0	0.778	0.723	50.1	0.628746
38	0	1	0	−1	0	0.648	0.641	55.1	0.69826
39	0	−1	0	1	0	0.748	0.743	70.5	0.272485
40	0	1	0	1	0	0.741	0.697	48.6	0.72102
41	0	0	0	0	0	0.755	0.745	57.5	0.413875
42	0	0	0	0	0	0.769	0.759	54.5	0.372734
43	0	0	0	0	0	0.745	0.735	57.3	0.447615
44	0	0	0	0	0	0.768	0.758	54.3	0.378996
45	0	0	0	0	0	0.743	0.733	55.4	0.442263
46	0	0	0	0	0	0.768	0.758	54.5	0.379459

**Table 3:** ANOVA for collector efficiency factor.

	Sum of		Mean	F	*p*-Value	
Source	Squares	Df	Square	Value	Prob > F	
Model	0.068	17	4.00 × 10^−3^	17.45	<0.0001	Significant
A-Mass flow rate	8.74 × 10^−3^	1	8.74 × 10^−3^	38.18	<0.0001	
B-Relative roughness pitch	0.016	1	0.016	69.61	<0.0001	
C-Relative roughness height	4.56 × 10^−3^	1	4.56 × 10^−3^	19.9	0.0001	
D- Re number	6.56 × 10^−3^	1	6.56 × 10^−3^	28.65	<0.0001	
E-Ambient temperature	4.80 × 10^−3^	1	4.80 × 10^−3^	20.94	<0.0001	
AC	2.89 × 10^−4^	1	2.89 × 10^−4^	1.26	0.2708	
AE	4.00 × 10^−4^	1	4.00 × 10^−4^	1.75	0.197	
BC	5.63 × 10^−5^	1	5.63 × 10^−5^	0.25	0.624	
BD	3.78 × 10^−3^	1	3.78 × 10^−3^	16.52	0.0004	
BE	8.10 × 10^−5^	1	8.10 × 10^−5^	0.35	0.5568	
CD	1.10 × 10^−4^	1	1.10 × 10^−4^	0.48	0.4935	
CE	4.90 × 10^−5^	1	4.90 × 10^−5^	0.21	0.6472	
A^2^	4.99 × 10^−3^	1	4.99 × 10^−3^	21.8	<0.0001	
B^2^	0.012	1	0.012	50.32	<0.0001	
C^2^	2.56 × 10^−4^	1	2.56 × 10^−4^	1.12	0.2993	
D^2^	7.74 × 10^−4^	1	7.74 × 10^−4^	3.38	0.0766	
E^2^	1.15 × 10^−3^	1	1.15 × 10^−3^	5.04	0.0328	
Residual	6.41 × 10^−3^	28	2.29 × 10^−4^			
Lack of Fit	5.69 × 10^−3^	23	2.47 × 10^−4^	1.71	0.2892	not significant
Pure Error	7.24 × 10^−4^	5	1.45 × 10^−4^			
Cor Total	0.074	45				

**Table 4:** ANOVA for collector efficiency factor.

	Sum of		Mean	F	*p*-Value	
Source	Squares	Df	Square	Value	Prob > F	
Model	0.096	17	$5.63\;\times \;10^{-3}$	18.35	<0.0001	Significant
A-Mass flow rate	0.017	1	0.017	54.64	<0.0001	
B-Relative roughness pitch	0.019	1	0.019	60.49	<0.0001	
C-Relative roughness height	$3.94\;\times \;10^{-3}$	1	$3.94\;\times \;10^{-3}$	12.83	0.0013	
D-Re number	0.012	1	0.012	39.42	<0.0001	
E-Ambient temperature	0.012	1	0.012	40.14	<0.0001	
AC	$1.32\;\times \;10^{-4}$	1	$1.32\;\times \;10^{-4}$	0.43	0.5169	
AD	$1.96\;\times \;10^{-4}$	1	$1.96\;\times \;10^{-4}$	0.64	0.4309	
BC	$4.20\;\times \;10^{-4}$	1	$4.20\;\times \;10^{-4}$	1.37	0.2518	
BD	$3.24\;\times \;10^{-4}$	1	$3.24\;\times \;10^{-4}$	1.06	0.313	
CD	$1.19\;\times \;10^{-3}$	1	$1.19\;\times \;10^{-3}$	3.88	0.0589	
CE	$1.02\;\times \;10^{-3}$	1	$1.02\;\times \;10^{-3}$	3.34	0.0784	
DE	$9.92\;\times \;10^{-4}$	1	$9.92\;\times \;10^{-4}$	3.23	0.083	
A^2^	$1.77\;\times \;10^{-3}$	1	$1.77\;\times \;10^{-3}$	5.77	0.0231	
B^2^	0.013	1	0.013	42.15	<0.0001	
C^2^	$1.96\;\times \;10^{-5}$	1	$1.96\;\times \;10^{-5}$	0.064	0.8022	
D^2^	$4.59\;\times \;10^{-4}$	1	$4.59\;\times \;10^{-4}$	1.49	0.2317	
E^2^	$5.56\;\times \;10^{-3}$	1	$5.56\;\times \;10^{-3}$	18.13	0.0002	
Residual	$8.59\;\times \;10^{-3}$	28	$3.07\;\times \;10^{-4}$			
Lack of Fit	$7.87\;\times \;10^{-3}$	23	$3.42\;\times \;10^{-4}$	2.36	0.1723	not significant
Pure Error	$7.24\;\times \;10^{-4}$	5	$1.45\;\times \;10^{-4}$			
Cor Total	0.1	45				

**Table 5:** ANOVA for Nusselt number.

	Sum of		Mean	F	*p*-Value	
Source	Squares	Df	Square	Value	Prob > F	
Model	1217.58	17	71.62	13.76	<0.0001	Significant
A-Mass flow rate	129.39	1	129.39	24.85	<0.0001	
B-Relative roughness pitch	279.73	1	279.73	53.73	<0.0001	
C-Relative roughness height	30.8	1	30.8	5.92	0.0216	
D-Re number	216.83	1	216.83	41.65	<0.0001	
E-Ambient temperature	57.38	1	57.38	11.02	0.0025	
AC	9.61	1	9.61	1.85	0.1851	
AD	84.64	1	84.64	16.26	0.0004	
AE	95.06	1	95.06	18.26	0.0002	
BC	22.09	1	22.09	4.24	0.0488	
BD	180.9	1	180.9	34.75	<0.0001	
BE	14.44	1	14.44	2.77	0.107	
CE	6	1	6	1.15	0.2921	
A^2^	68.93	1	68.93	13.24	0.0011	
B^2^	19.47	1	19.47	3.74	0.0633	
C^2^	1.65	1	1.65	0.32	0.5774	
D^2^	0.82	1	0.82	0.16	0.6947	
E^2^	8.62	1	8.62	1.66	0.2088	
Residual	145.78	28	5.21			
Lack of Fit	135.13	23	5.88	2.76	0.1312	not significant
Pure Error	10.65	5	2.13			
Cor Total	1363.36	45				

**Table 6:** ANOVA for Closeness factor.

	Sum of		Mean	F	*p*-Value		
Source	Squares	Df	Square	Value	Prob > F		
Model	1.46	20	0.073	29.37	<0.0001	Significant	
A-Mass flow rate	0.2	1	0.2	82.65	<0.0001		
B-Relative roughness pitch	0.34	1	0.34	136.68	<0.0001		
C-Relative roughness height	0.053	1	0.053	21.33	0.0001		
D-Re number	0.2	1	0.2	82.56	<0.0001		
E-Ambient temperature	0.1	1	0.1	41.05	<0.0001		
AB	$1.21\;\times \;10^{-5}$	1	$1.21\;\times \;10^{-5}$	4.89 × 10^−3^	0.9448		
AC	$5.12\;\times \;10^{-3}$	1	$5.12\;\times \;10^{-3}$	2.07	0.163		
AD	0.043	1	0.043	17.53	0.0003		
AE	0.032	1	0.032	13.07	0.0013		
BC	$9.42\;\times \;10^{-3}$	1	$9.42\;\times \;10^{-3}$	3.8	0.0625		
BD	0.036	1	0.036	14.49	0.0008		
BE	$8.31\;\times \;10^{-3}$	1	$8.31\;\times \;10^{-3}$	3.35	0.0791		
CD	$4.76\;\times \;10^{-3}$	1	$4.76\;\times \;10^{-3}$	1.92	0.1781		
CE	$6.52\;\times \;10^{-4}$	1	$6.52\;\times \;10^{-4}$	0.26	0.6125		
DE	$1.74\;\times \;10^{-3}$	1	$1.74\;\times \;10^{-3}$	0.7	0.4105		
A^2^	0.12	1	0.12	48.37	<0.0001		
B^2^	0.31	1	0.31	125.3	<0.0001		
C^2^	0.013	1	0.013	5.16	0.032		
D^2^	$1.51\;\times \;10^{-4}$	1	$1.51\;\times \;10^{-4}$	0.061	0.8072		
E^2^	$6.14\;\times \;10^{-4}$	1	$6.14\;\times \;10^{-4}$	0.25	0.623		
Residual	0.062	25	$2.48\;\times \;10^{-3}$				
Lack of Fit	0.056	20	$2.82\times \;\;10^{-3}$	2.49	0.1579	not significant	
Pure Error	$5.65\;\times \;10^{-3}$	5	$1.13\;\times \;10^{-3}$				
Cor Total	1.52	45					

**Table 7:** Optimum solutions and confirmative test results obtained through CSO algorithm.

Process Variables	A	B	C	D	E	TOSIS Based CSO	TOSIS CF	Expt CF	% Improvement
Optimum Result						1.46	0.919987	0.99898	8.58%

## Data Availability

Not applicable.
